# Evaluating Mycelium as an insulation material: A comparative study on thermal performance, comfort, and energy efficiency

**DOI:** 10.12688/f1000research.162989.2

**Published:** 2026-01-24

**Authors:** Bassant Khaled, Nermine Hany, Gihan Mosaad

**Affiliations:** 1 Department of Architectural and Engineering Environmental Design, Arab Academy for Science Technology and Maritime Transport, Alexandria Governorate, 1029, Egypt

**Keywords:** Mycelium Insulation, sustainable building material, energy efficiency, thermal comfort

## Abstract

**Background:**

Since the building sector contributes significantly to carbon emissions, using sustainable materials is more crucial than ever. Mycelium is investigated in this study as a natural substitute for conventional insulation materials like rock wool and XPS. Its thermal performance has been evaluated in a residential building in New Cairo, Egypt, using Design-Builder simulations, paying particular attention to U-values, discomfort hours, PPD-PMV, and energy consumption. The results demonstrate that mycelium is environmentally safe, biodegradable, and provides insulation that is comparable to XPS. This demonstrates its promise as a sustainable option for building in the future.

**Methods:**

This research uses Design-Builder software version 7.3.0.046 integrated with Energy-Plus to simulate the thermal conductivity of mycelium compared to two of the most frequently utilized traditional materials for insulation in Egypt, in Janna Compound, New Cairo. The analysis examines energy consumption, thermal comfort, Predicted Mean Vote (PMV), and Predicted Percentage of Dissatisfaction (PPD%). The study provides a comparative evaluation of mycelium insulation compared to traditional materials like XPS and Rockwool.

**Results:**

According to the simulation results, mycelium insulation performs comparably to XPS regarding U-values, discomfort hours, PPD-PMV, and energy consumption. Specifically, mycelium achieved a 0.323 U-value, reduced discomfort hours percentage to 16.9%, and achieved a ratio of energy reduction of 15.8% compared to the base case. These results demonstrate how mycelium has the potential to compete with traditional insulation materials while offering significant sustainability advantages, such as biodegradability and a lower carbon footprint.

**Conclusion:**

In Janna Compound, New Cairo, the research shows how mycelium insulation can improve thermal comfort and save energy usage. It performs similarly to XPS but has more positive environmental effects. These results support the integration of mycelium into Egypt’s sustainable housing practices, providing key insights for architects, developers, and policymakers focused on energy-efficient and sustainable urban development.

## Introduction

The main concern nowadays worldwide is the problem of energy consumption in building sectors, which approximately reaches 40% of the total energy used. ^1^ In Egypt over 50% of energy consumption is for residential buildings. ^2^ As energy demand continues to rise, finding sustainable solutions to lower energy consumption in buildings has become critical. ^2^ As energy demand continues to rise, finding sustainable solutions to reduce energy consumption and associated carbon emissions has become critical. Improving the thermal performance of the building envelope, particularly external walls which contribute 25–30% of total heat gains, is one of the most effective strategies for enhancing energy efficiency and reducing cooling loads. ^3^

### Research problem

**Figure 1.**
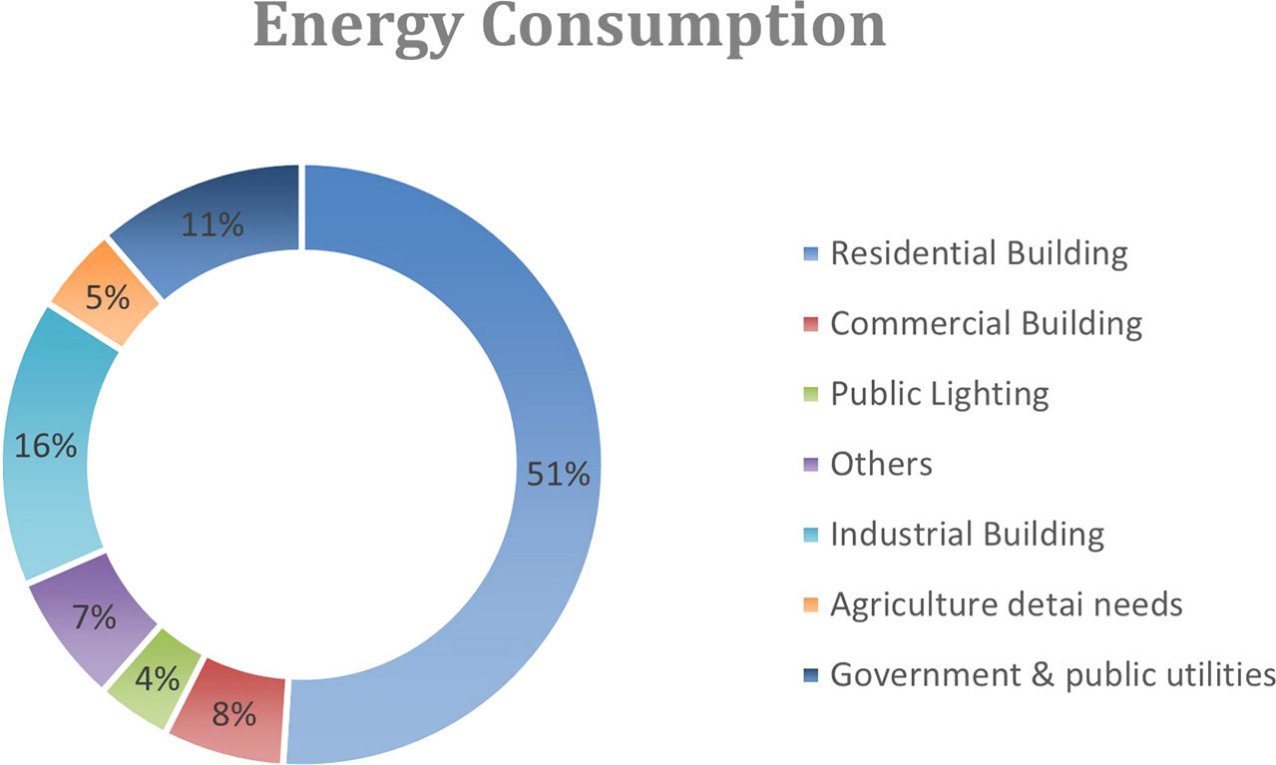
Egypt energy consumption graph.

Worldwide, the construction sector stands as a major energy user, responsible for consuming more than 40% of the total energy supply, with an average annual increase of 1.5% from 2012 to 2040. ^1^ In Egypt, due to the rapid growth and higher living standards, residential buildings consume over half of the country’s electricity ^2^ as shown in Figure 1.

Each year in the housing sector, energy use increased by 7%, and without finding a solution, the consumption could be more than double by 2030, increasing from 60 to 135 million tons of oil equivalent (MTOE). ^2^ This problem not only strains energy resources but also increases the environmental challenges, making energy efficiency a critical priority.

One of the main approaches to reducing energy consumption in the building sector and to meet with Egypt’s Vision 2030, is through the improvement of insulation materials. Studies show that enhancing insulation in walls, roofs, and windows could decrease CO _2_ emissions by 2190 kilotons per year and save up to 842MW of peak energy demand. ^1^ Alongside decreasing energy consumption, this approach safeguards the planet while promoting healthier and more sustainable living conditions for people. At the same time, Egypt generates an estimated 30–35 million tonnes of agricultural waste annually, much of which is burned or discarded. ^4^ This highlights the potential of bio-based alternatives, such as mycelium composites, which can valorize local agricultural residues while reducing environmental impact.

This research explores mycelium as a sustainable and circular insulation material. It first examines its properties and potential as an alternative to conventional insulation. Then, it compares mycelium to commonly used materials in Egypt, such as XPS and Rockwool. Finally, a simulation on Janna Compound analyses four scenarios, evaluating energy consumption, discomfort hours, PMV, and PPD to assess mycelium’s feasibility in building insulation.

### Research aim

The purpose of this research is to increase academics’ and designers’ awareness of the benefits and potential of mycelium insulation. It investigates how well mycelium insulation performs in comparison to two commonly used insulation materials and proposes it to be a sustainable alternative for the construction sector in Egypt.

## Methods

The research focuses on examining the performance of mycelium insulation, utilizing a residential building prototype in a typical floor of a type A building within Janna Compound as the case study, located in New Cairo, Egypt. A literature review and a base case study are the two primary elements of the research.

### Literature review

The literature review will address several important subjects, including the definition of mycelium, and explore its sustainability and circularity as a material. It will also illustrate its thermal conductivity properties, which are essential for evaluating its thermal performance as an insulation material. Following this, the review will provide an overview of two traditional insulation materials that are commonly used in Egypt, XPS, and Rockwool.

### Base case study

The research’s second section examines a case study of a typical floor in a Type A building within Janna Compound, New Cairo. This simulates how insulating materials affect thermal comfort and energy usage, including Predicted Mean Vote (PMV), Predicted Percentage of Dissatisfaction (PPD%), and thermal comfort hours. The primary aim is to assess how well mycelium insulation performs in comparison to commonly used materials like XPS and Rockwool, to evaluate its usefulness. The scenarios evaluated are:

• **Baseline model:** Wall without insulation.
• **Scenario 1:** Wall insulated with mycelium.
• **Scenario 2:** Wall insulated with XPS.
• **Scenario 3:** Wall insulated with Rockwool.

The investigation’s methodology evaluates mycelium’s efficacy in comparison to two commonly used materials to improve residential buildings’ thermal comfort in Egypt. Design-Builder version 7.3.0.046 & Energy-Plus plugin software were used for this simulation, which will evaluate discomfort hours, PMV-PPD, and energy consumption for a typical floor in a Type A building in Janna Compound, emphasizing how these materials and techniques affect the thermal condition.

## Literature review

**Figure 2.**
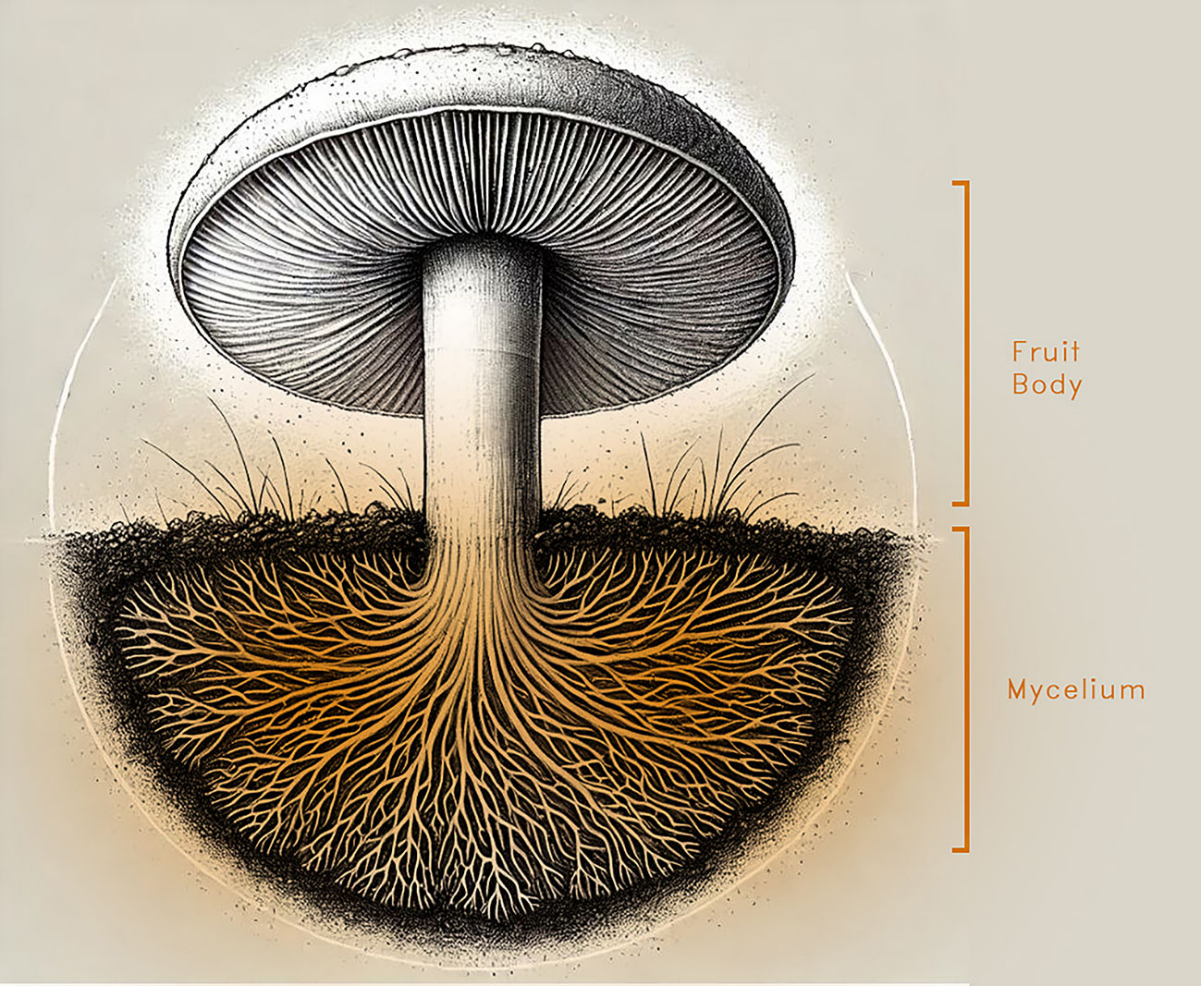
The fungi split into underground mycelium and a fruiting body, created using AI tools and modified in Photoshop.

Mycelium is the vegetative structure of fungi, consisting of a dense, root-like network of fine filaments called hyphae. These hyphae typically range from 1 to 30 micrometres in diameter and spread through the substrate, binding organic matter together. ^5^ This underground network supports the growth of the visible fruiting body (mushroom) as shown in Figure 2.

In recent years, mycelium has gained a lot of attention from academic and industrial sectors because, during growth, it requires a minimal amount of energy, lacks by-product generation, and has multiple potential uses. ^6^

Indoor air quality was enhanced by using mycelium, due to its natural air-purifying properties, especially in filtering particulate matter (PM). Studies have demonstrated its effectiveness due to its breathable structure, which helps capture and remove airborne such as PM2.5 and PM10. Leading to improving air purity. ^7^

Recent studies have demonstrated the potential of mycelium-based composites in building applications, particularly regarding thermal conductivity, biodegradability, and low embodied energy. ^8^^,^^9^ Additionally, mycelium has the capability to capture and store carbon during growth. ^10^ Mycelium-based materials have a variety of applications across various industries, they can be utilized as acoustic panels, tiles, bricks, and furniture due to their lightweight and permeable structure, as well as their thermal, fire, and water resistance, acoustic absorption capabilities, and aesthetic appeal. ^11^

### Sustainability and circularity of mycelium material

**Figure 3.**
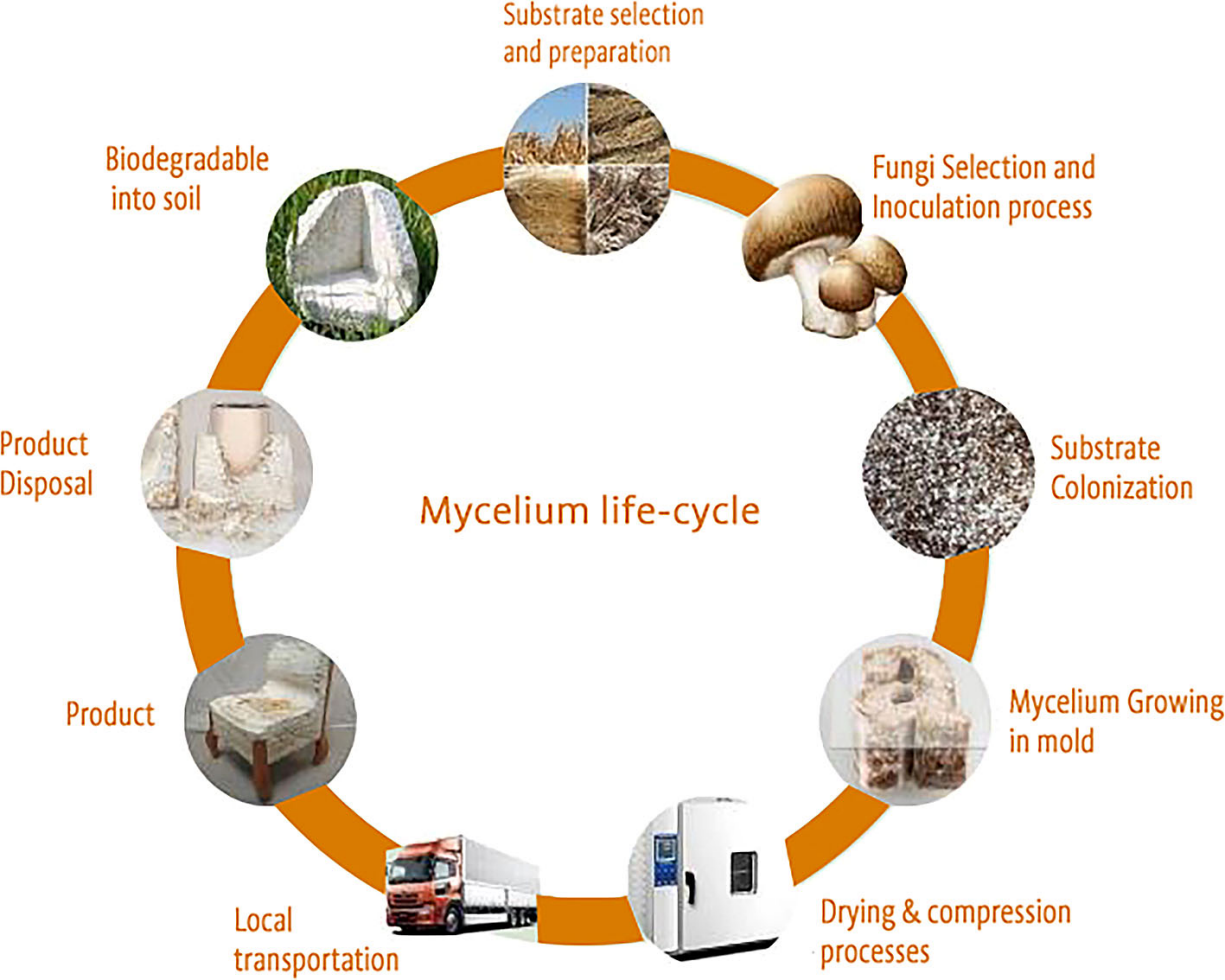
The sustainable life cycle of materials sourced from mycelium.

The environmentally friendly characteristics of mycelium-based materials and their potential role in shaping the future of sustainable construction. Six principal factors support construction ^12^ as shown in Figure 3.

  -Cost-effectiveness and abundant raw materials   -Biodegradability

  -Rapid manufacturing process          -Flexibility

  -Minimal Energy consumption         -Cardle-to-cradle life cycle

In Egypt, mycelium production remains limited but is gradually expanding, mainly in the Cairo–Giza region and parts of the Nile Delta. Commonly cultivated species such as oyster (Pleurotus ostreatus), button (Agaricus bisporus), and shiitake (Lentinula edodes) can also be used for composite production. ^13^ Local initiatives demonstrate this potential: Cupmena ( https://cupmena.com/) repurposes spent coffee grounds for mushroom cultivation and bio-fertilizer, ^14^^,^^15^ while Mycelium Egypt ( https://mycellium.co/) is developing biomaterials from agricultural residues for construction and packaging. ^16^ These efforts show that mycelium-based materials can be produced locally using abundant agricultural waste resources, reducing reliance on imports and supporting circular economy goals.

### Thermal and physical properties of mycelium insulation material

Heat transfer, heat storage capacity, material density, and thermal diffusivity are among the thermal and physical attributes of insulation materials used in the construction sector. These features define a material’s efficiency in terms of heat absorption, transport, and retention. When combined, these characteristics help a structure use less energy and maintain a pleasant interior temperature, which lowers the need for artificial heating or cooling. ^17^

When combining mycelium with agricultural residences, it stands out as a promising alternative to foam insulation materials. This enables the formation of porous composites, such as foams, to be formed. Due to their higher moisture content, the samples show a higher Heat transfer rate. ^18^ Thermal conductivity refers to how efficiently a material transfers heat; lower values indicate better insulation performance. Research on Mycelium-based composites suggests that their thermal conductivity ranges between 0.029-0.104 W/mK, making them a good option for insulation. ^19^

Table 1 shows the characteristics of the mycelium insulation panel made by BIOHM ^®^ ( https://www.biohm.co.uk/), which is used in the simulation, the panel dimension is 1.2×2.4m, with a thickness of 0.075m. ^20^

**Table 1.** Performance characteristics of the Mycelium Insulation panel used in the simulation. ^20^

Insulation material	Density (kg/m ^3^)	Thermal conductivity (W/mK)	Compressive strength (MPa)	Thickness (mm)
**Mycelium**	128	0.03	0.12	75

**Table 2.** Insulation properties of XPS and Rockwool. ^24, 25^

Insulation material	Density (kg/m ^3^)	Thermal conductivity (W/mK)	Compressive strength (MPa)	Thickness (mm)
**XPS**	28-40	0.028-0.031	0.25-0.45	70
**Rockwool**	40-120	0.035-1.36	0.14	50

### Selection criteria of the traditional insulation materials

Eight factors were taken into consideration while choosing materials from the Egyptian market: fire resistance, simplicity of application, cost, absorption, pressure force, durability, water vapor transmission, and thermal conductivity. ^21^

To determine how mycelium would function, the simulation compares its thermal performance with two conventional materials that are utilized in Egypt.

• XPS (extruded polystyrene) is a closed-cell foam, giving it greater roughness, stiffness, and thermal resistance. Its low water vapor permeability also makes it well suited for use in humid environments. ^22^
• Rockwool insulation material is made by heating natural stones like dolomite, basalt, and diabase to extremely high temperatures (1400-1600°C) and transformed into fibers. These fibers are then bound together using binders like starch, oil, or resins. ^23^

An Egyptian company specializing in building insulation materials, Rockal Al Alamia ( https://www.rockalinsulation.com/), for Insulation, provided the data shown in Table 2. Since the simulation and the materials are based in Egypt, the selection of this data source is consistent with the study’s setting and provides relevance to regional environmental conditions and construction methods. According to the company’s profile, Rockal is a leading Egyptian producer and supplier of building insulation products, including extruded polystyrene (XPS) and Rockwool, which are widely available and used in the local construction market. ^26^

## Case study

Egypt’s residential buildings heavily rely on air conditioning, increasing energy costs. ^1^ This research compares mycelium insulation with two commonly used materials in Egypt, such as XPS and Rockwool, on a typical floor of a Type A building in Janna Compound to assess its potential as a sustainable alternative in reducing energy consumption and enhancing thermal efficiency.

### Thermal comfort and bioclimatic chart analysis in New Cairo, Egypt

**Figure 4.**
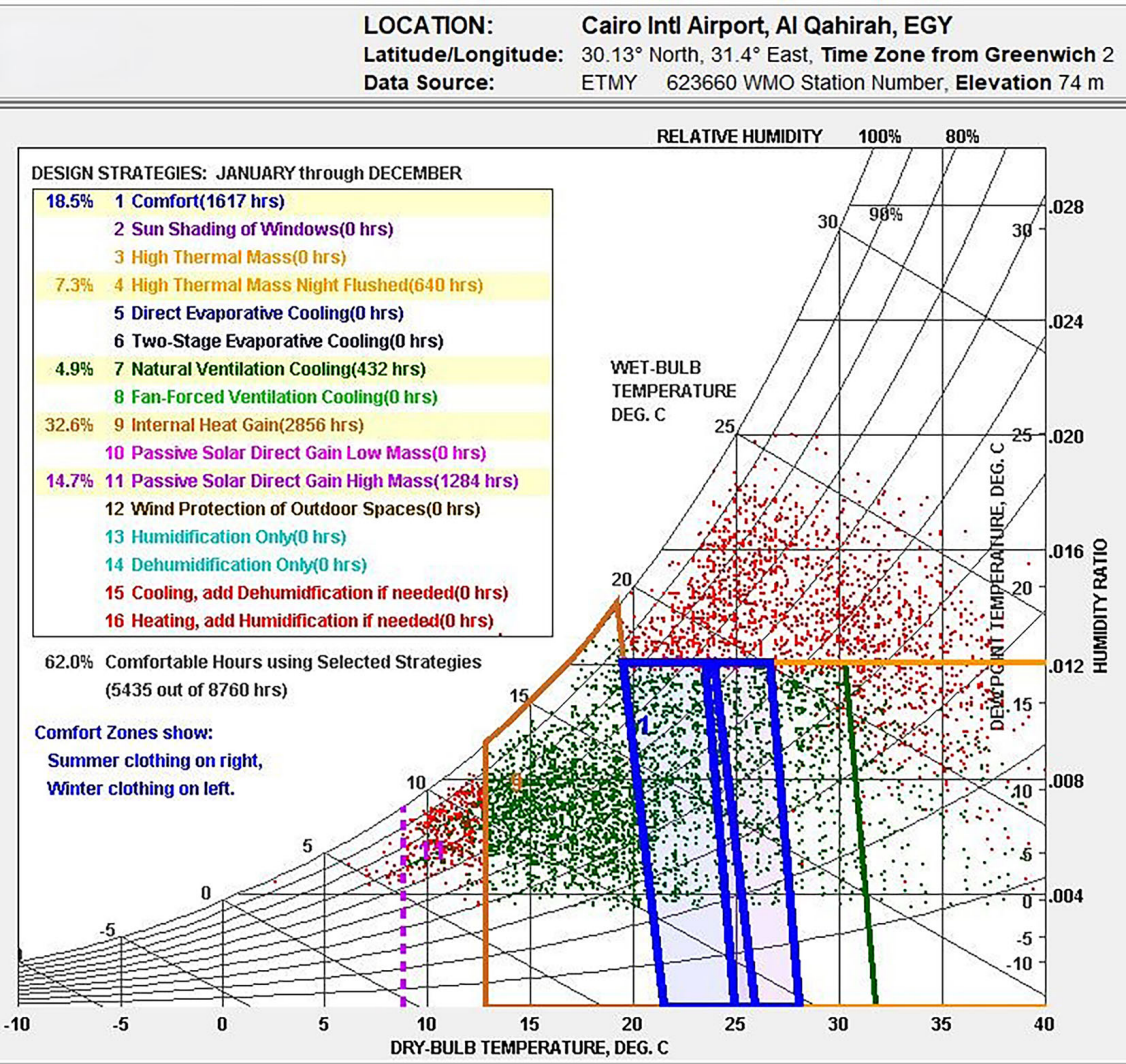
Bioclimatic Analysis of Egypt's climate using Climate Consultant, indicating 62% comfortable hours (Red) with passive.

This research examines the bioclimatic conditions in Egypt utilizing Climate Consultant v.6.0 to create a psychrometric chart based on weather data. This chart aids in identifying effective design strategies aimed at improving indoor thermal comfort, as illustrated in Figure 4, which showcases climate-responsive design methods.

Figure 4 reveals that only 18.5% annually falls within Egypt’s thermal comfort range. Several climate-responsive design strategies were evaluated to improve thermal comfort:

• Internal heat gains could increase comfortable hours by 32.6%, particularly in winter.
• 4.9% of thermal comfort was attributed to natural ventilation and cooling.
• Night ventilation, when paired with high thermal mass, enhanced comfort by 7.3%.
• The integration of high thermal mass and passive solar heating boosts comfort by 14.7%, resulting in 62% of the time feeling comfortable.

Nevertheless, the results are based on general climate data recommendations. To identify the most effective solutions, further investigation of the building’s design parameters will be required.

### Overview of New Cairo, Egypt’s Climate

New Cairo, which is located between latitudes 30.03°N and longitudes 31.47°E, has a mainly hot desert climate. Climatic data indicates that summer temperatures typically reach between 35–45°C in August, highlighting the need for effective cooling systems during the hot months. In contrast, winter temperatures are lower, ranging from 15–20°C, which may reduce heating requirements. ^27^

### Case study description of Janna Compound, New Cairo

**Figure 5.**
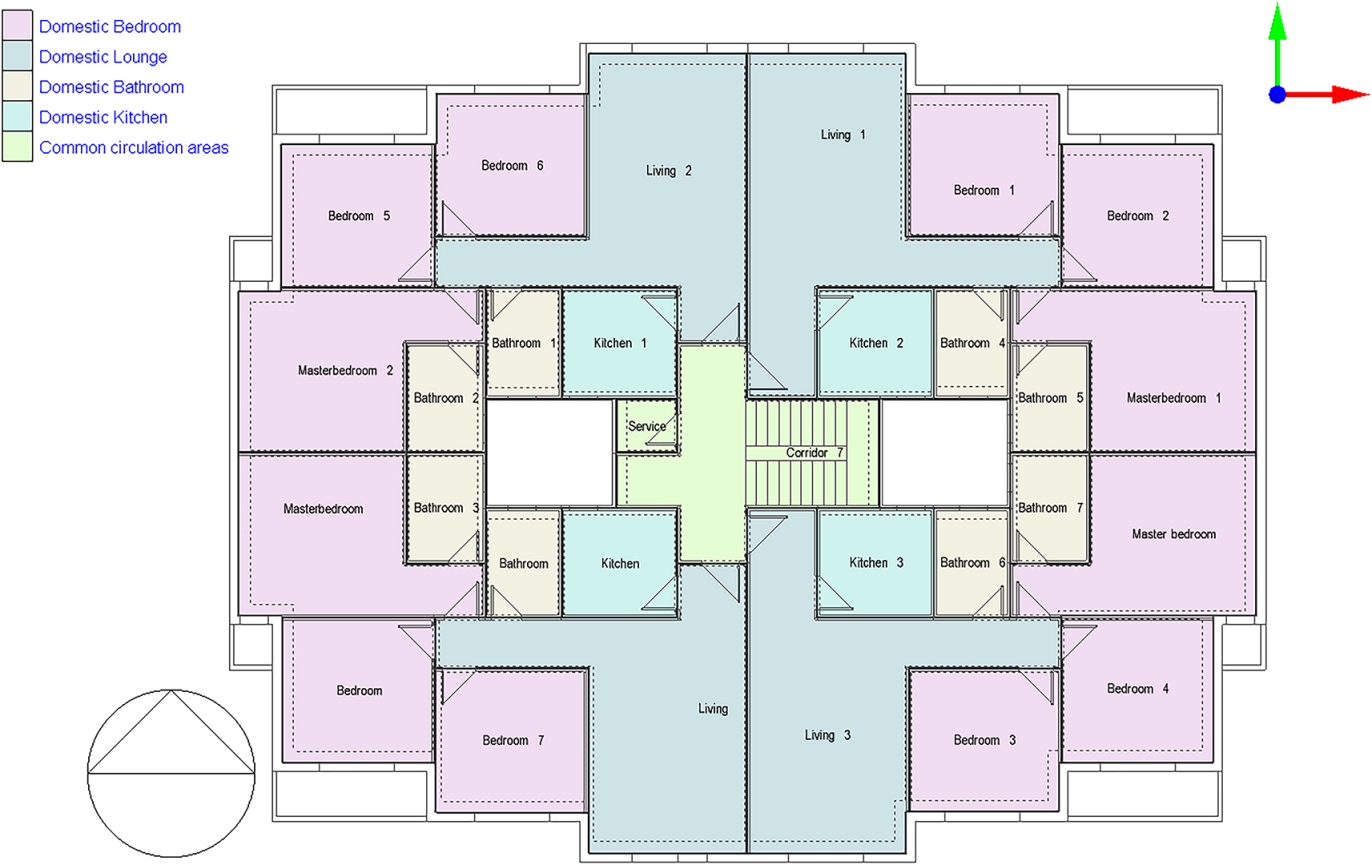
Type A building plan, typical floor of Janna Compound, New Cairo, Egypt.

The examined building model is a type A building, part of a residential project created by the Egyptian Government to accommodate the growing need for homes. It’s a six-story building, each containing four residential apartments, each around 130 square meters. Each flat typically houses at least two residents, and the simulation was conducted on a typical floor of the building ^28^ as shown in Figure 5.

**Figure 6.**
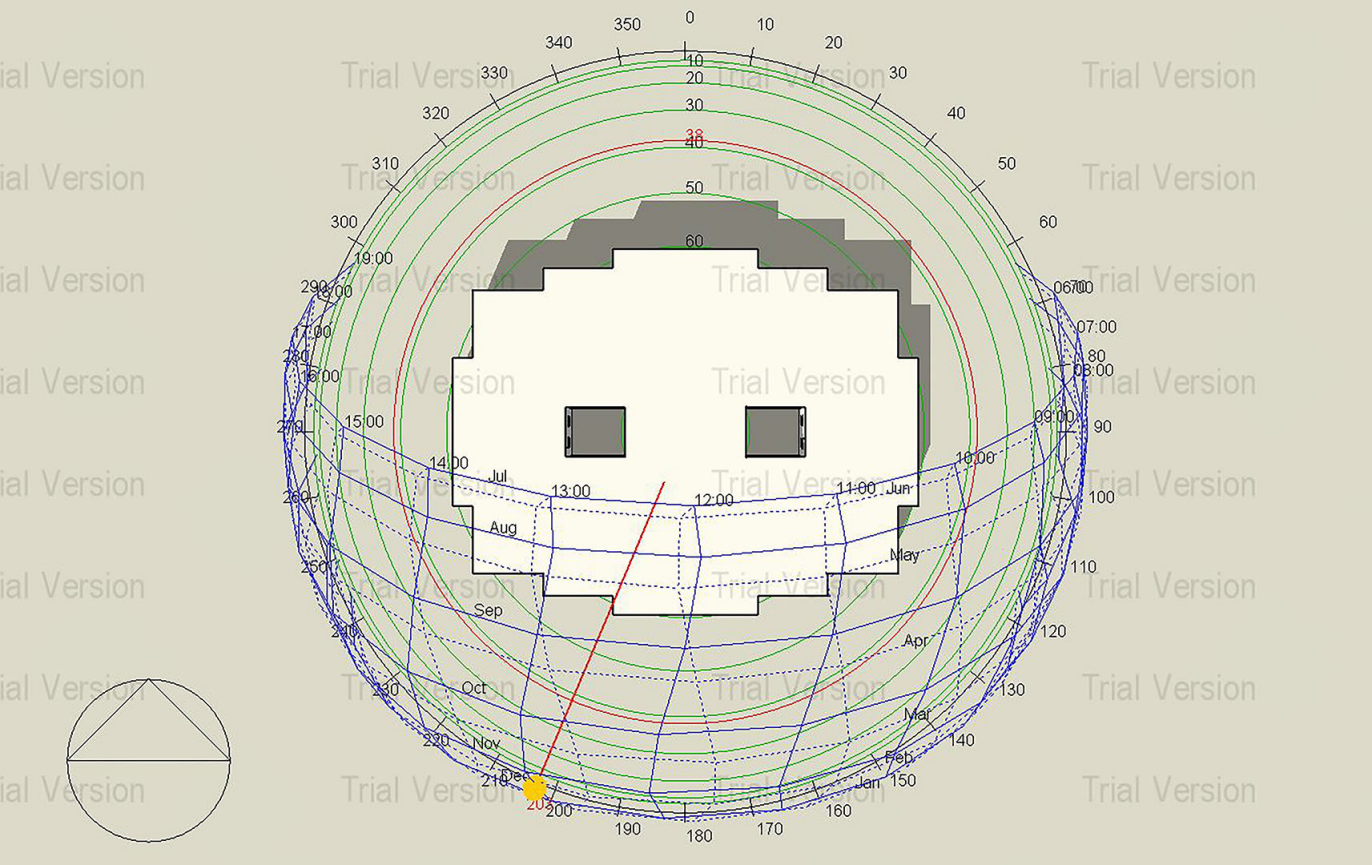
Sun path Diagram of the building, Janna compound.

The typical floor plan has four similar apartments, each covering 130 sqm. Each apartment includes a kitchen, living room, two bathrooms, and three bedrooms ^28^ as shown in Figure 5.

### Design builder project file configuration for Janna Compound case study

Janna Compound is a residential gated compound developed by the Egyptian Government to accommodate the growing need for homes across multiple cities across Egypt, including El-Sheikh Zayed, Al-Minya, New Cairo, and Mansura. The project consistently utilizes the same design models (Types A and B), regardless of local climate variations or material costs. ^29^ The Type A building in New Cairo, Egypt, was selected for simulation to compare Mycelium insulation with traditional materials, as many residential projects in the area lack appropriate insulation, making this comparison crucial for enhancing energy efficiency and thermal comfort. As shown in Figure 6, solar exposure on the selected building provided by the sun path diagram is a key factor in thermal performance assessment.

### Data entry

Design Builder 7.0.2.006 with an Energy Plus 9.4 plugin is the energy simulation software used in this case study. It works by modeling the building’s environmental conditions on an annual and monthly basis, including humidity, lighting, thermal balance, and energy consumption.

*Activity*

The activity of each space is specified accurately as well as the number of users, occupancy density, and metabolic rate as shown in Table 3.

**Table 3.** Specifications for Janna Compound activity in design builder [By the researchers].

Activity	
Template	*Domestic Circulation – Residential Spaces*
Number of Users	*2 users minimum*
Occupancy Density (people/m ^2^)	*0.117 people/m ^2^*
Metabolic Rate	*0.925 (Assuming 2 adults, 1 man and 1 woman)*

*Construction*

Accurate simulation requires detailed building information, encompassing the materials used for external walls, slabs, and roofs. The U-value and insulation properties play a critical role in energy consumption, as they affect thermal exchange. Table 4 outlines the layers of building envelope materials utilized in the Design-Builder program.

**Table 4.** Construction layers of the base case in design builder [By the researchers].

*Construction*	*Layers of the building envelope (Thickness, m)*	
External wall	The Outermost Layer (External Plaster) 0.005 Cement/plaster/mortar-plaster 0.02 Brickwork Outer 0.25 Cement/plaster/mortar-plaster 0.02 Innermost Layer (Acrylic Paint) 0.001	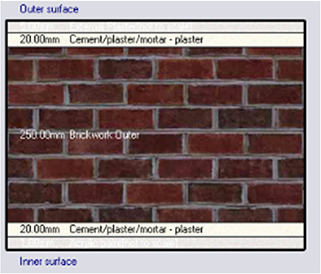
Internal partition	Gypsum Plaster 0.0012 Brickwork inner 0.12 Gypsum Plaster 0.0012	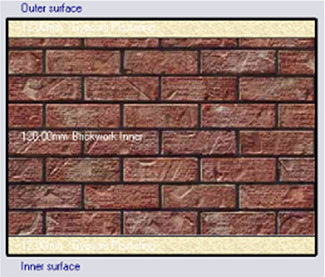
Roof	Cement/plaster/mortar-plaster 0.02 Concrete 0.07 EPS thermal insulation layer 0.05 Reinforced concrete 2% steel 0.15 Cement/plaster/mortar-plaster 0.02	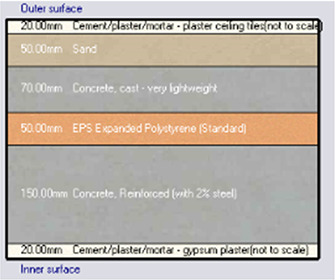
Floor	Ceramic tiles 0.02 Cement layer 0.02 Sand 0.07 Reinforced concrete 2% steel 0.15 Cement/plaster/mortar-plaster 0.02	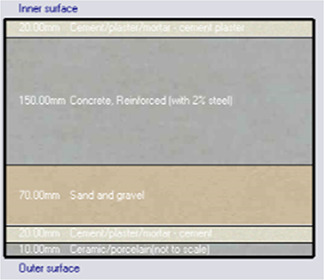

*Openings*

Energy consumption is greatly affected by thermal gain and loss via windows, particularly in hot regions where solar thermal energy plays a significant role. For accurate simulation, detailed information on window glazing and shading systems is essential, as shown in Table 5.

**Table 5.** Selected opening materials in design builder [By the researchers].

Openings	
Internal Door	*Plywood Lightweight, 0.05m thickness, 0.9 W, 2.2 H*
Windows	*Single glazing, clear, no shading on windows, Aluminum frame*

*HVAC*

HVAC was employed to calculate total energy consumption during the hot months to assess how mycelium insulation would reduce energy usage. However, for the calculation of PMV, PPD, and discomfort hours, the HVAC was turned off. The HVAC configurations utilized in the program are presented in Table 6 below.

**Table 6.** HVAC settings designated in design builder [By the researchers].

HVAC	
HVAC Template	*Split unit + Natural Ventilation*
Cooling System COP	*1.80*
Heating System COP	*0.85*
Natural Ventilation	*Checked on*

### Case study simulation findings

The simulation was conducted from the beginning of January to the end of December, evaluating four different cases of different insulation materials, as summarized in Table 7. However, when calculating PMV, PPD, and energy consumption, the simulation focused primarily on the hot months, from June 1 to August 31.

• Base Case: Brick wall without any insulating layers.
• Alternative-1: Mycelium Insulation
• Alternative-2: XPS-Extruded polystyrene Insulation
• Alternative-3: Rock wool Insulation

**Table 7.** Simulation four scenarios to be tested in design builder [By the researchers].

Case	Insulation type	
Base Case	No Insulation	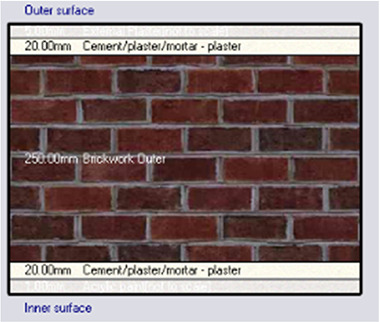
	*Outermost layer: External Plaster 0.005m* *Cement/plaster/mortar-plaster 0.020m* *Brickwork Outer 0.250m* *Cement/plaster/mortar-plaster 0.020m* *Innermost layer: Acrylic 0.001m*	
Alternative-1	Mycelium	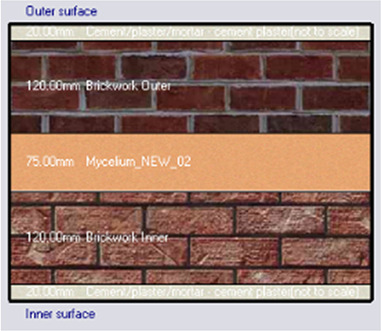
	*Outermost layer: External Plaster 0.005m* *Cement/plaster/mortar-plaster 0.020m* *Brickwork Outer 0.120m* *Mycelium Insulation panel 0.075m* *Brickwork Inner 0.120m* *Cement/plaster/mortar-plaster 0.020m* *Innermost layer: Acrylic 0.001m*	
Alternative-2	XPS-Extruded polystyrene	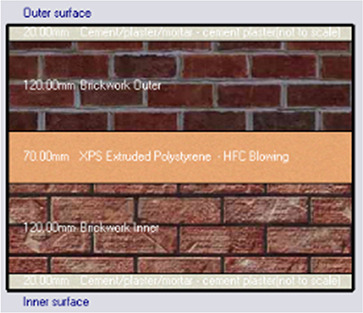
	*Outermost layer: External Plaster 0.005m* *Cement/plaster/mortar-plaster 0.020m* *Brickwork Outer 0.120m* *XPS Insulation panel 0.070m* *Brickwork Inner 0.120m* *Cement/plaster/mortar-plaster 0.020m Innermost layer: Acrylic 0.001m*	
Alternative-3	Rockwool	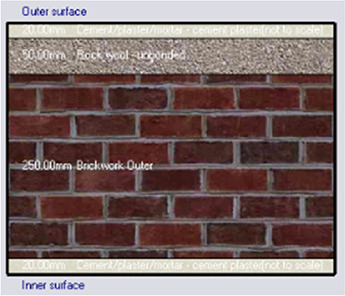
	*Outermost layer: External Plaster 0.005m* *Rockwool 0.050m* *Cement/plaster/mortar-plaster 0.020m* *Brickwork Outer 0.250m* *Cement/plaster/mortar-plaster 0.020m* *Innermost layer: Acrylic 0.001m*	

**Figure 7.**
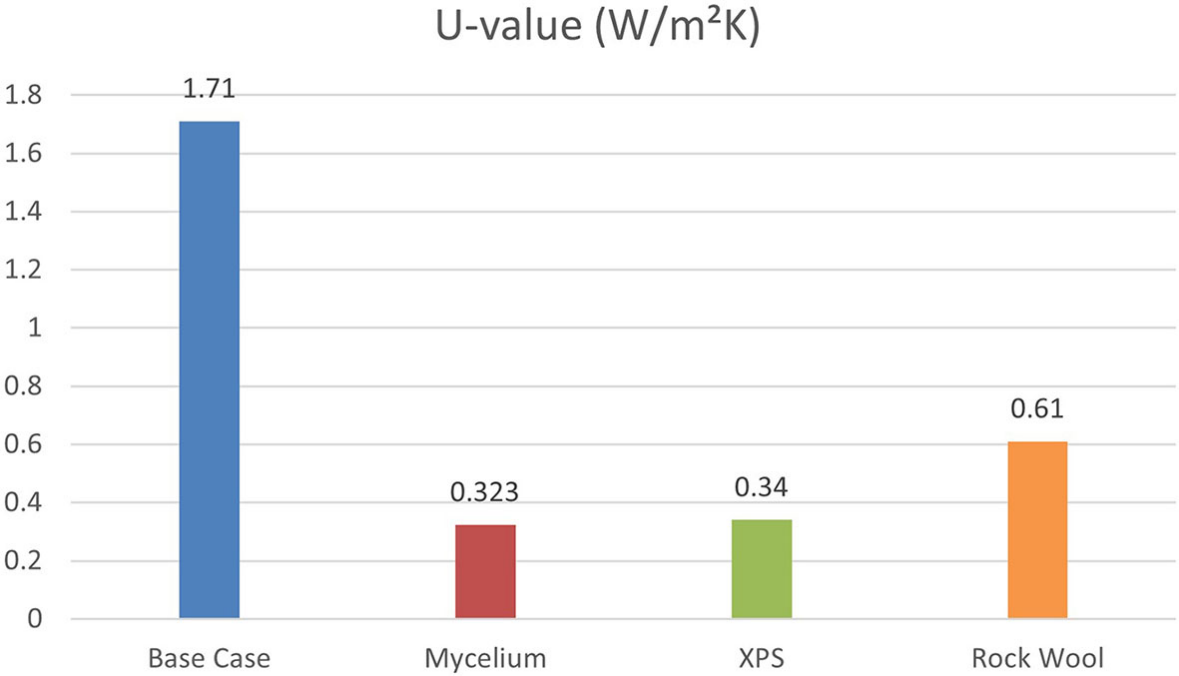
Thermal transmittance (U-Value) for the Four Scenarios.

Energy-efficient building design focuses on managing exterior thermophysical properties, such as the U-value (thermal transmittance), which is key for assessing thermal efficiency and energy savings. Figure 7 shows that the base case without insulation has the highest U-value at 1.71 W/m ^2^K, while Mycelium insulation has the lowest at 0.323 W/m ^2^K. XPS and Rock Wool have U-values of 0.34 W/m ^2^K and 0.61 W/m ^2^K, respectively, indicating varying levels of thermal performance. Therefore, Mycelium demonstrates the best U-value compared to other traditional materials.

**Figure 8.**
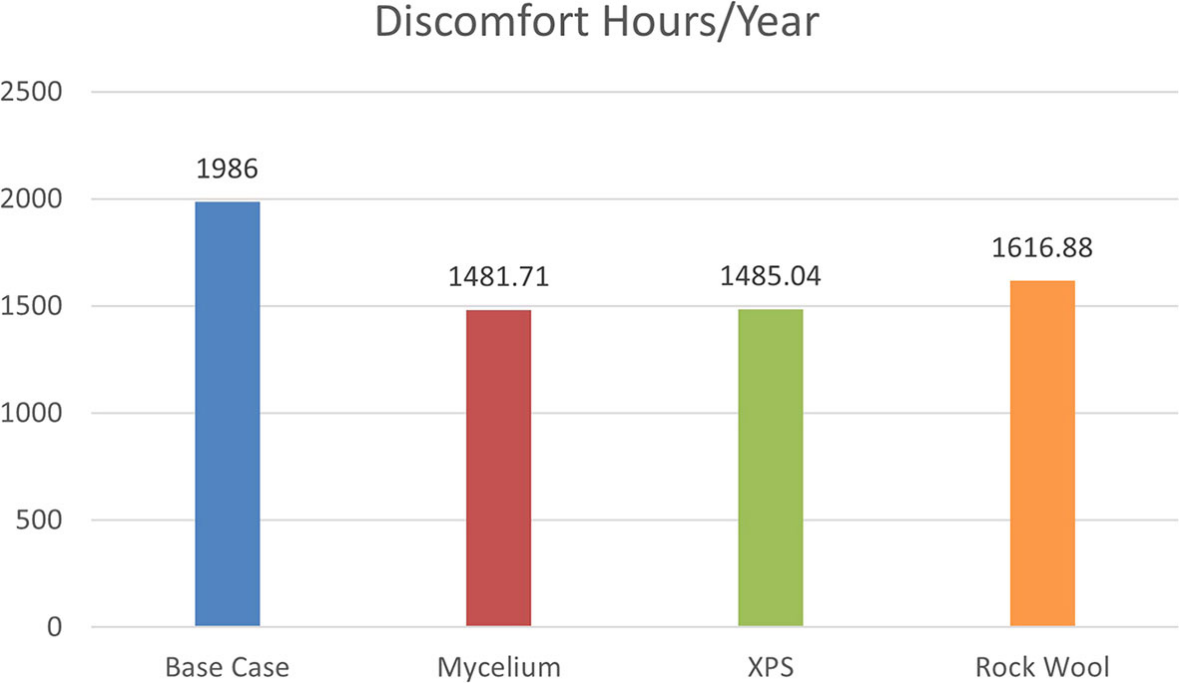
Discomfort hours/year for the four different scenarios.

The simulation, based on ASHRAE 55–2004 standards, evaluates discomfort hours due to inadequate cooling, heating, and humidity levels without HVAC systems. As shown in Figure 8, the base case with a conventional wall recorded 1986 discomfort hours annually. Mycelium insulation reduced this to 1481.71 hours, while XPS reduced it to 1485.04 hours, and Rock Wool to 1616.88 hours. The results show that Mycelium performs similarly to XPS, indicating that it can compete with traditional materials in terms of thermal comfort.

**Figure 9.**
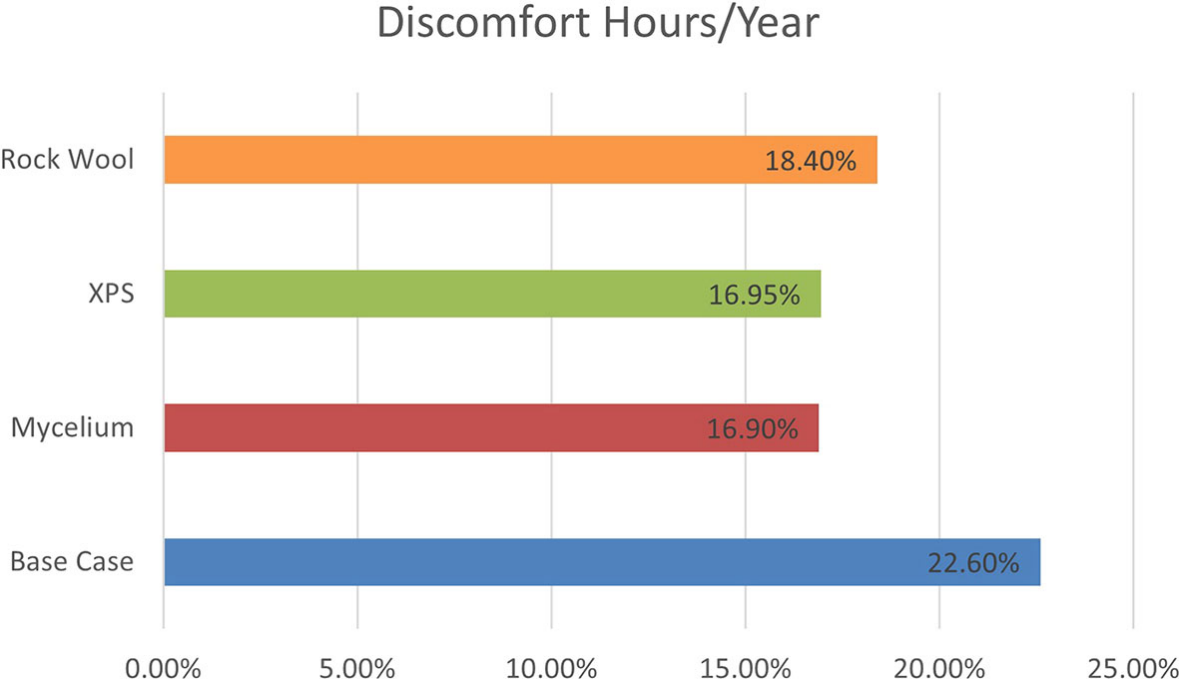
Percentage of Discomfort hours/year for the four different scenarios.

The percentage of discomfort hours per year for the four scenarios is as follows: the base case recorded 22.6% discomfort, while Mycelium showed 16.9%, XPS recorded 16.95%, and Rock Wool had 18.4% discomfort. These results demonstrate that Mycelium not only performs better than Rock Wool but also shows a similar percentage of discomfort hours to XPS, making Mycelium an equally effective choice for improving thermal comfort and energy efficiency. As shown in Figure 9.

**Figure 10.**
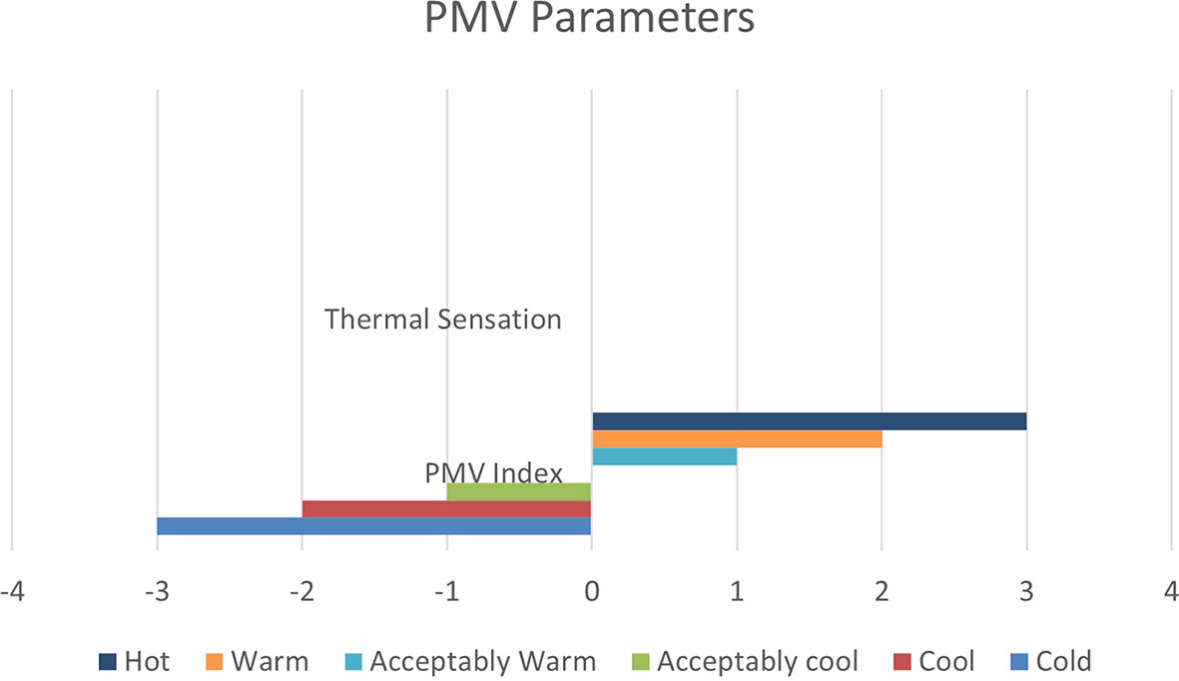
PMV (Predicted Mean Vote) Scaling Factors.

From June 1 to September 30, specific thermal comfort strategies are applied to enhance comfort during the summer and minimize air conditioning use. The evaluation focuses on Predicted Mean Vote (PMV) and Percentage of People Dissatisfied (PPD%), using a PMV scale from -3 (cool) to +3 (hot). By concentrating on the hot months, as shown in Figure 10, this approach allows for precise adjustments to environmental systems, ensuring optimal thermal comfort and energy efficiency during peak cooling periods.

Table 8 illustrates the variations in the outcomes relative to the baseline case across all four scenarios, emphasizing the PPD% values during the warmest months.

**Table 8.** PMV-PPD% in hot months for the four scenarios in design builder [By the researchers].

Scenarios	Max Fanger PMV (Hot months)	Max Fanger PPD% (Hot months)
Base Case	*1.46*	*53.26%*
Alternative-1	*1.37*	*50.48%*
Alternative-2	*1.37*	*50.46%*
Alternative-3	*1.35*	*49.76%*

**Table 9.** Comparison between all scenarios results [By the researchers].

Comparison between the four scenarios				
Scenario	Base case 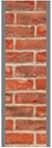	Mycelium 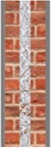	XPS 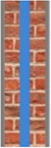	Rock wool 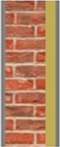
U-Value	1.71	0.323	0.34	0.61
% of Discomfort hours/year	22.6%	16.9	16.9	18.4
% of Energy reduction	-	15.8	15.7	13.3
PMV	1.46	1.37	1.37	1.35

**Figure 11.**
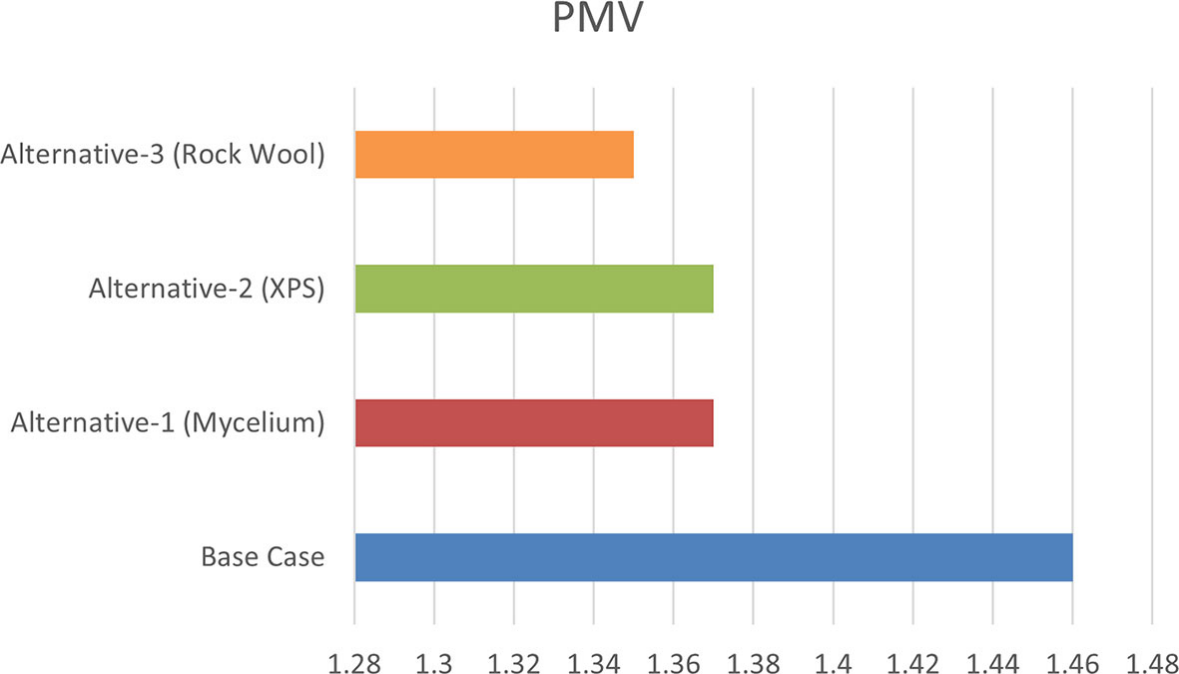
PMV (Predicted Mean Vote) for the Four Simulation Scenarios in Design Builder.

**Figure 12.**
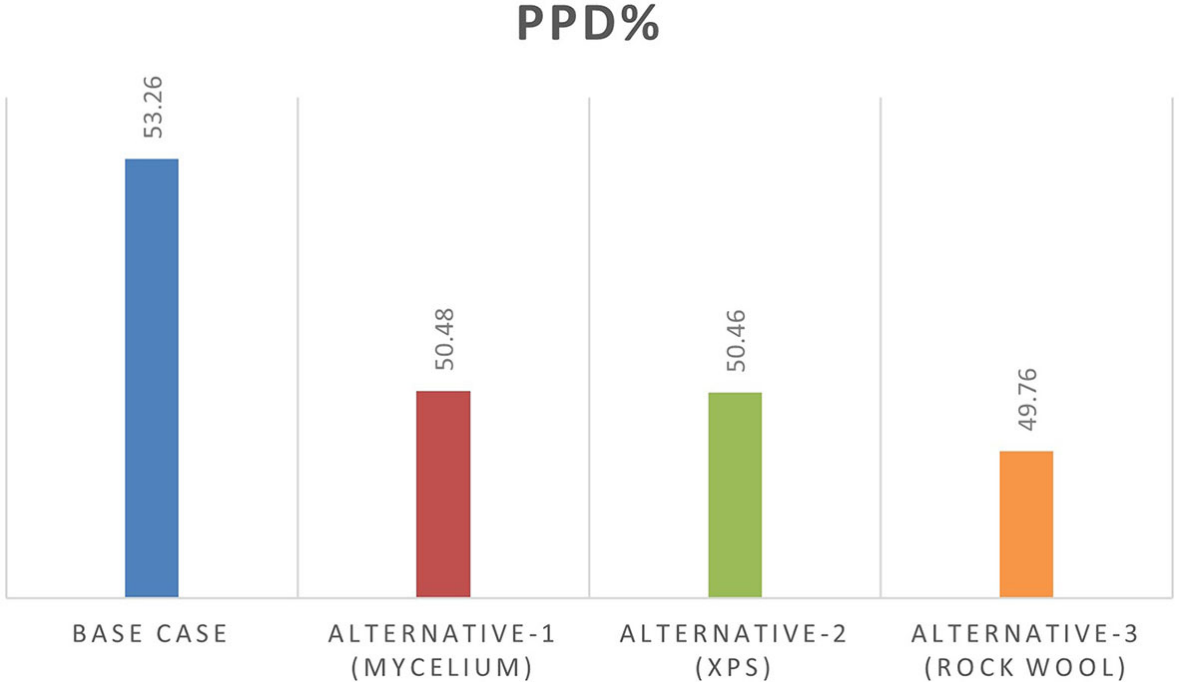
PPD% (Predicted Percentage of Dissatisfaction) for the Four Simulation Scenarios in Design Builder.

As shown in Table 9, the comparison of the four scenarios in the case study offers key insights for improving thermal comfort. In the base case, without insulation, the Predicted Mean Vote (PMV) is 1.46, with a Percentage of People Dissatisfied (PPD) of 53.26%. Adding Mycelium insulation in Alternative-1 improves the PMV to 1.37 and reduces the PPD to 50.48%. Alternative-2, which uses XPS insulation, shows similar results, with a PMV of 1.37 and a PPD of 50.46%. Alternative-3, featuring Rock Wool insulation, slightly improves comfort further, achieving a PMV of 1.35 and a PPD of 49.76%. As shown in Figures 11 and 12.

Mycelium, with its impressive performance values, not only matches traditional insulation materials in terms of thermal efficiency but also provides added advantages. As a sustainable and biodegradable material, Mycelium is an eco-friendly choice, making it a highly suitable option for thermal insulation.

**Figure 13.**
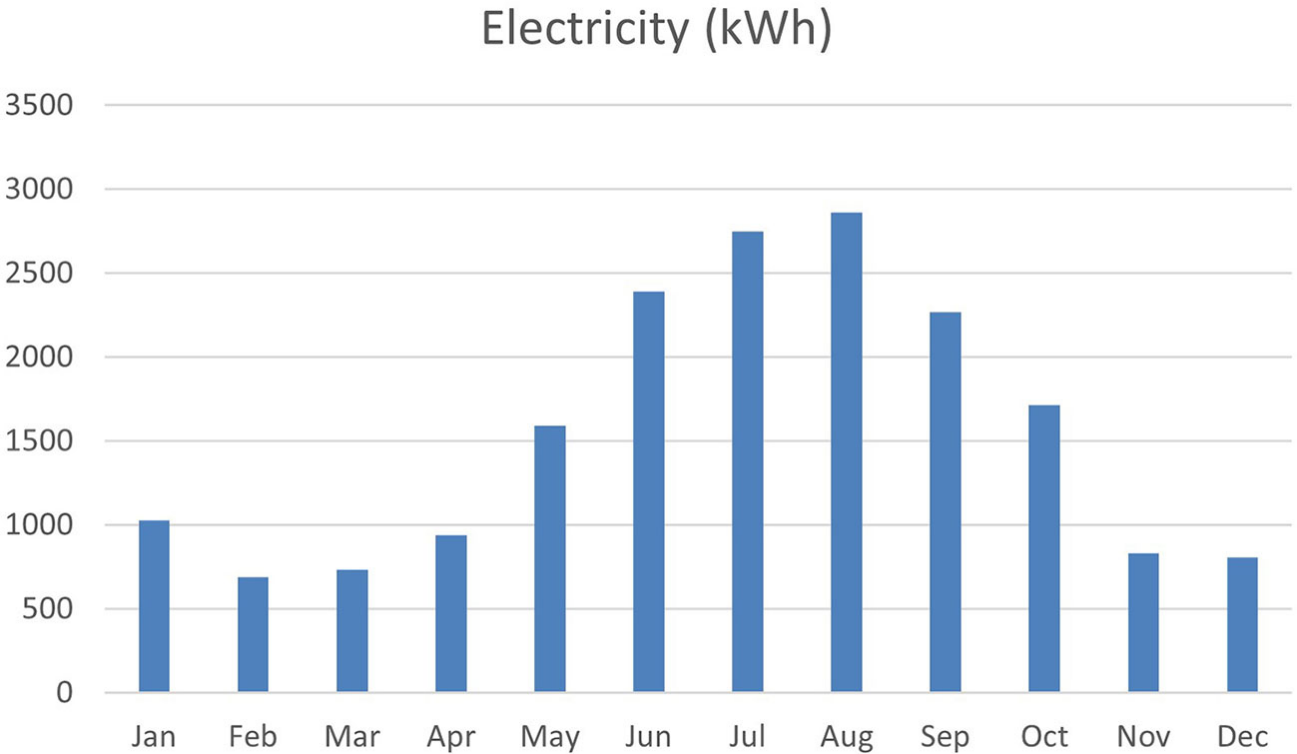
Energy consumption for the baseline case from design builder.

The simulation of annual energy consumption reveals that electricity usage varies throughout the year, with a notable increase during the summer months. In contrast, consumption is relatively low during the cooler months, as heating and cooling needs are reduced. However, from May to August, energy consumption peaks due to the heightened cooling demands driven by intense solar radiation. As shown in Figure 13, this simulation underscores the significant effect of seasonal changes on overall energy performance.

**Figure 14.**
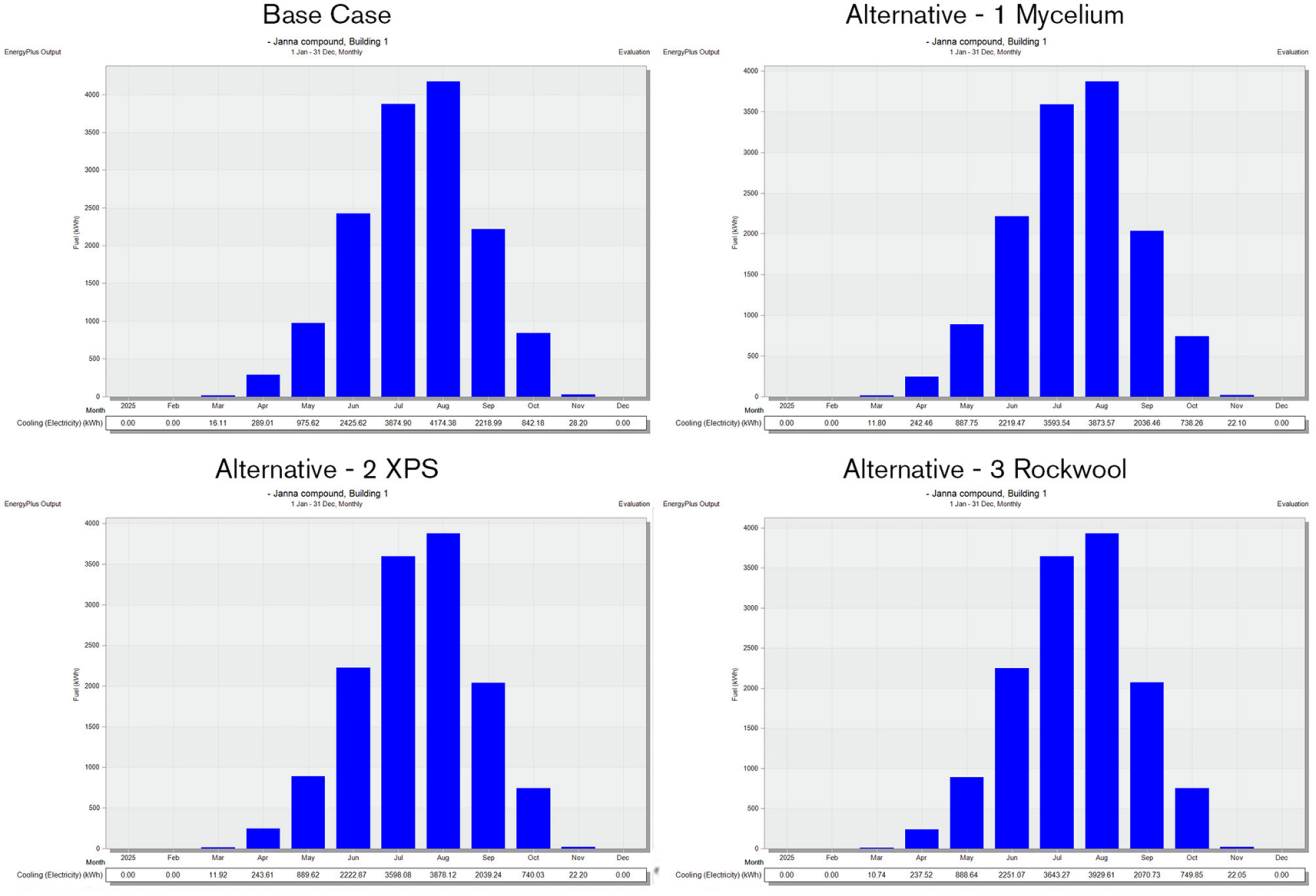
Energy consumption for the 4 scenarios in hot months in design builder.

Regarding energy consumption, it is observed that the base case wall material led to higher energy usage during the warmer months. Particularly from May to August, due to increased solar radiation. The simulation findings indicate that the total electricity consumption for the base case during these hot months was 10,203.75 kWh. In comparison, XPS insulation resulted in 8,601.1 kWh, Mycelium insulation consumed 8,587.42 kWh, and Rock Wool insulation had a consumption of 8,838.77 kWh. As shown in Figure 14.

**Figure 15.**
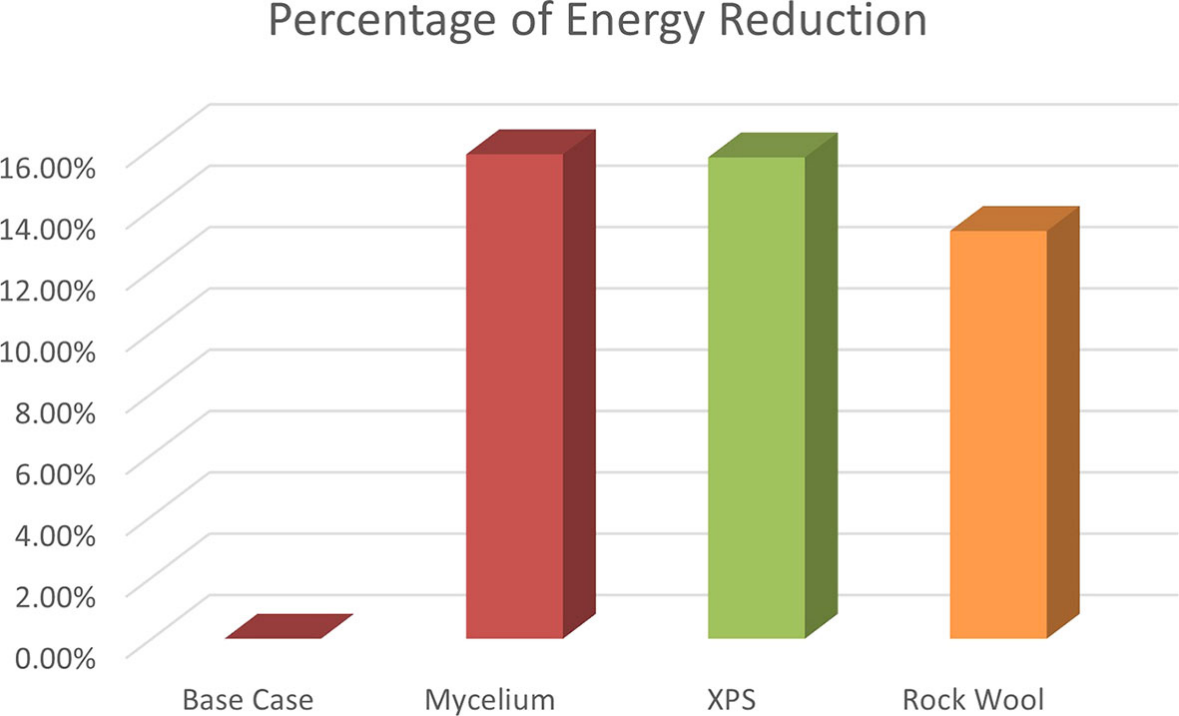
Percentage of energy reduction for the 4 scenarios in hot months in design builder.

The energy consumption reductions are 15.7% for XPS, 15.8% for Mycelium, and 13.3% for Rock Wool. As shown in Figure 15. These results show that Mycelium performs similarly to XPS in terms of energy savings and makes a notable contribution to improving energy efficiency. According to these results, CO _2_ emission was also reduced, Mycelium reduced 979.5 kg of CO _2_, while XPS reduced 971.2 kg and Rock wool reduced 827.18 kg of CO _2_.

To calculate the percentage of energy reduction, the energy consumption of the base case scenario (without intervention) is compared to the improved scenario (with intervention). The formula used is:

$$\text{Percentage of Energy Reduction}=\left(\frac{\text{Base case Energy Consumption}-\text{Improved Energy Consumption}}{\text{Base case Energy Consumption}}\right)\times 100$$

## Conclusions and recommendations

The simulation was performed on a typical floor of a Type A building in New Cairo’s Janna compound. Each floor had four apartments, each approximately 130 m ^2^. The research compared Mycelium insulation to traditional materials like XPS and Rock Wool in terms of their thermal performance and energy efficiency.

The results showed that Mycelium insulation performed effectively, with a PPD of 50.8% and a PMV of 1.37. This result was much better than the base case and equivalent to XPS (PPD = 50.46%, PMV = 1.37). In terms of U-value, Mycelium demonstrated the best performance with a U-value of 0.323 W/m ^2^K, compared to XPS at 0.34 W/m ^2^K and Rock Wool at 0.61 W/m ^2^K. Regarding energy consumption, Mycelium reduced energy usage by 15.8%, which was better than Rock wool (13.3%), and comparable to XPS (15.7%). These results highlight Mycelium as a sustainable and energy-efficient alternative to traditional insulation materials.

In conclusion, Mycelium and XPS have nearly the same values. However, XPS (Extruded polystyrene) is well-known and widely used because of its affordability, availability, and strong thermal performance. Despite this, XPS has several disadvantages: it requires high energy input during manufacturing, releases toxic gases during production, produces dense smoke and hazardous emissions when exposed to fire, and is unstable under UV radiation. ^30^

In Contrast, Mycelium Insulation offers a sustainable and circular alternative with several key advantages: ^12^

• Cost-effectiveness: Mycelium grows using agricultural waste, making it an inexpensive and available resource
• Biodegradability: Mycelium naturally decomposes at the end of its life cycle
• Rapid manufacture: it can be cultivated in a few weeks
• Flexibility: it can be molded into different shapes and densities
• Minimal Energy Consumption: Growing mycelium required less energy compared to other materials
• Cradle-to-cradle life cycle: Mycelium fits with the circularity idea, it can be composed and reintegrated into the natural ecosystem with no harm to the environment

To provide a clearer comparison between mycelium and XPS, Table 10 summarises key performance and sustainability indicators based on published literature.

**Table 10.** Comparison between Mycelium Insulation and XPS [By the researchers].

Insulation material	Mycelium	XPS	References
**Thermal conductivity**	0.03(W/mK)	0.028-0.031(W/mK)	[ 20][ 25]
**Energy**	Growing at room temperature using biological processes	Energy-intensive manufacturing	[ 31][ 32]
**Emission**	Very low carbon emission (-244 g CO _2_eq/m ^3^)	High carbon emissions (271.8 kg CO _2_eq/m ^3^)	[ 33]
**Env. Impact**	Low environmental footprint, grown from agricultural waste using minimal energy	High environmental impact, made from fossil fuels (petrochemicals)	[ 18][ 34]
**Biodegradability**	Fully biodegradable and compostable	Remain in landfills for hundreds of years	[ 18][ 35]
**Fire Safety**	Naturally fire-resistant, Chars but does not release toxic fumes	Highly flammable unless treated release toxic smoke when burned	[ 36][ 37]

Given the positive results of this research, it is essential to further explore how Mycelium can be integrated into the construction industry and its potential for large-scale implementation. The following recommendations aim to guide future efforts in adopting Mycelium as a mainstream insulation material.

• Further Research: To examine the longevity and economic feasibility of mycelium in various climate conditions and building types.
• Adoption by the Construction sector: Encourage the use of Mycelium in construction, especially in regions where traditional materials contribute to environmental pollution.
• Regulatory Support: Advocate for policies that promote the use of sustainable, non-toxic insulation materials like Mycelium, which can significantly contribute to energy-efficient buildings and the reduction of carbon footprints.

## Ethics and consent

Ethical approval and consent were not required.

## Data Availability

Zenodo: Evaluating Mycelium as an Insulation Material: A Comparative Study on Thermal Performance, Comfort, and Energy Efficiency. https://doi.org/10.5281/zenodo.15020538 ^38^ This project contains the following underlying data
1. Egypt Energy Consumption Graph (Illustrates the percentage distribution of energy consumption across various sectors) 2. The Fungi Structure (Illustrates the structure of the mushroom, underground mycelium, and the fruit body created using AI tools and modified in photoshop) 3. The sustainable life cycle of materials sourced from mycelium (shows the sustainable life cycle of mycelium products) 4. Bioclimatic Analysis of Egypt’s climate Using Climate Consultant (shows climatic data such as temperature patterns, humidity levels, and solar radiation across Egypt.) 5. Type A Building Plan, Typical Floor of Janna Compound, New Cairo, Egypt (Case study typical floor plan, designed in design builder) 6. Sun path Diagram of the building, Janna compound (Sun path diagram in Janna compound) 7. Thermal transmittance (U-Value) for the Four Scenarios 8. Discomfort hours/year for the four different scenarios 9. Percentage of Discomfort hours/year for the four different scenarios 10. PMV (Predicted Mean Vote) Scaling Factors 11. PMV (Predicted Mean Vote) for the Four Simulation Scenarios in Design Builder 12. PPD% (Predicted Percentage of Dissatisfaction) for the Four Simulation Scenarios in Design Builder 13. Energy Consumption for the Baseline Case from Design Builder 14. Energy Consumption for the 4 Scenarios in Hot Months in Design Builder 15. Percentage of Energy Reduction for the 4 Scenarios in Hot Months in Design Builder 16. Excel file for Cairo Weather Data (The same weather file was used in the design builder for simulation data) 17. Janna Compound, New Cairo (Two design builder files include the 3D model and the simulation one with HVAC and one without) Data are available under the terms of the Creative Commons Attribution 4.0 International license (CC-BY 4.0).

## Software availability statement

Design-Builder v7.3.0.046 ( https://designbuilder.co.uk/) was used for energy simulation modeling. This is proprietary software, however, an open-access alternative such as OpenStudio can achieve similar outcomes.

EnergyPlus ( https://energyplus.net/) is an open-access tool used in this research.
