# Supplementary material for: Evaluating Mycelium as an insulation material: A comparative study on thermal performance, comfort, and energy efficiency
Source: F1000Res. 2026 Sep 21;14:459. Originally published 2025 Apr 24. [Version 3] doi: 10.12688/f1000research.162989.3 (PMC12954402; doi:10.12688/f1000research.162989.3)
Supplement: Peer review history

# Reviewer report: Reviewer response for version 2

**DOI:** 10.5256/f1000research.193600.r471892

**Published:** 2026-04-16

**Authors:** Rouwaida Hussein Ali

**Affiliations:** 1 University of Diyala, Diayla, Iraq

This manuscript addresses a **highly relevant and timely topic** in sustainable construction by investigating **mycelium-based insulation** as an alternative to conventional materials (XPS and Rockwool). The study contributes to the growing body of research on **bio-based materials in hot climates**, particularly within the Egyptian context.

 The integration of **DesignBuilder + EnergyPlus simulation** and the focus on **thermal comfort (PMV/PPD), U-value, and energy consumption** are appropriate and aligned with current research practices.

 However, despite its potential, the manuscript requires **major revisions** before it can meet indexing standards due to issues in:

- Methodological rigor
- Reproducibility
- Depth of analysis
- Strength of evidence

The study exhibits several important limitations that reduce its scientific rigor and reliability.

 First, it relies entirely on simulation using DesignBuilder/EnergyPlus without any experimental validation, which limits confidence in the results and their real-world applicability.

 In addition, the methodology lacks sufficient transparency, as key simulation parameters such as HVAC setpoints, internal loads, infiltration rates, and occupancy schedules are not clearly reported, making the study difficult to reproduce.

 Furthermore, the comparison between insulation materials is not fully consistent, since different thicknesses were used (e.g., 75 mm for mycelium vs. 70 mm for XPS and 50 mm for Rockwool), which may bias the results in favor of certain materials .

 The analytical depth is also limited, as the study does not include statistical validation, sensitivity analysis, or uncertainty assessment, despite reporting very small performance differences between materials.

 Another key weakness is the over-reliance on literature to support environmental and sustainability claims without conducting a dedicated life cycle assessment (LCA) or cost analysis within the study itself.

 Finally, the literature review is relatively limited and lacks sufficient engagement with recent high-impact studies, while some sections of the manuscript (particularly the introduction and conclusions) remain descriptive and insufficiently supported by strong scientific evidence.

Is the case presented with sufficient detail to be useful for teaching or other practitioners?

Partly

Is the work clearly and accurately presented and does it cite the current literature?

Partly

If applicable, is the statistical analysis and its interpretation appropriate?

Yes

Are all the source data underlying the results available to ensure full reproducibility?

Yes

Are the conclusions drawn adequately supported by the results?

Partly

Is the background of the case’s history and progression described in sufficient detail?

Partly

Reviewer Expertise:

engineering

I confirm that I have read this submission and believe that I have an appropriate level of expertise to confirm that it is of an acceptable scientific standard, however I have significant reservations, as outlined above.

# Reviewer report: Reviewer response for version 2

**DOI:** 10.5256/f1000research.193600.r457749

**Published:** 2026-03-02

**Authors:** Hamid Yusoff

**Affiliations:** 1 Universiti Teknologi MARA, Shah Alam, Malaysia

1. Summary of the Article

 This manuscript evaluates mycelium-based insulation as a sustainable alternative to conventional insulation materials (XPS and Rockwool) in residential buildings located in New Cairo, Egypt. The study uses Design-Builder integrated with Energy Plus to simulate thermal performance for a typical floor of a Type A building in Janna Compound.

 Four wall configurations are modeled:

- Base case (no insulation)
- Mycelium insulation
- XPS insulation
- Rockwool insulation

The comparison includes:

- U-value (thermal transmittance)
- Annual discomfort hours
- PMV (Predicted Mean Vote)
- PPD (Predicted Percentage of Dissatisfied)
- Energy consumption during hot months
- CO₂ emission reductions

The authors conclude that mycelium performs comparably to XPS in thermal performance and energy savings while offering environmental advantages such as biodegradability and lower embodied carbon.

 The topic is highly relevant and contributes to the growing interest in bio-based materials for sustainable construction in hot climates. However, several methodological clarifications and evidentiary improvements are required before the conclusions can be considered fully supported.

 2. Major Comments (Must Be Addressed)

 A. Justification of Insulation Thickness and Wall Assembly

 The materials are compared using different insulation thicknesses:

- Mycelium: 75 mm
- XPS: 70 mm
- Rockwool: 50 mm

Since insulation thickness directly affects U-value and energy performance, this difference may influence the results.

**Required action:**

- Either standardize thickness across all materials, or
- Clearly justify the selection (e.g., equivalent R-values, commercial availability, Egyptian market norms, or structural constraints).

Additionally, Rockwool is placed differently in the wall assembly compared to mycelium and XPS. The reasoning for this difference must be explained and supported by construction practice references.

 Without this clarification, the comparison risks methodological bias.

 B. Insufficient Detail in Simulation Parameters

 The simulation settings lack sufficient transparency for full reproducibility.

 The manuscript does not clearly specify:

- Cooling and heating setpoints
- Internal loads (lighting, equipment gains)
- Infiltration rates (ACH)
- Detailed occupancy schedules
- Control strategies for natural ventilation

**Required action:**

 Provide a comprehensive summary table of all simulation assumptions and parameters. This is essential for scientific rigor and reproducibility.

 C. Limited Statistical or Sensitivity Analysis

 The study reports performance differences, but:

- No sensitivity analysis is conducted.
- No uncertainty analysis is presented.
- The differences between mycelium and XPS are very small (e.g., 15.8% vs 15.7% energy reduction) yet are discussed as meaningful.

**Required action:**

 At minimum:

- Clarify whether these differences are practically significant.
- Include a brief discussion on modeling uncertainty.
- Consider performing a sensitivity analysis (e.g., varying thermal conductivity values).

Without this, claims of superiority or equivalence should be more cautiously framed.

 D. Environmental Claims Require Stronger Quantification

 The manuscript emphasizes sustainability advantages of mycelium (low embodied energy, carbon sequestration, biodegradability). While references are included, the study itself does not perform:

- A lifecycle assessment (LCA)
- A cost analysis
- A durability comparison

Therefore, the argument for adoption remains partly speculative.

**Required action:**

- Clearly distinguish between simulation-based findings and literature-based sustainability claims.
- Avoid overstating environmental benefits unless supported by quantified analysis within this study.

Is the case presented with sufficient detail to be useful for teaching or other practitioners?

Partly

Is the work clearly and accurately presented and does it cite the current literature?

Partly

If applicable, is the statistical analysis and its interpretation appropriate?

Not applicable

Are all the source data underlying the results available to ensure full reproducibility?

Yes

Are the conclusions drawn adequately supported by the results?

Partly

Is the background of the case’s history and progression described in sufficient detail?

Partly

Reviewer Expertise:

1. Mycelium composite

I confirm that I have read this submission and believe that I have an appropriate level of expertise to confirm that it is of an acceptable scientific standard, however I have significant reservations, as outlined above.

# Response

**Published:** 2026-09-02

**Authors:** passant khaled

**Affiliations:** Interior, Arab Academy for Science Technology and Maritime Transport College of Engineering and Technology, Alexandria, Alexandria Governorate, Egypt

Response to Comment A – Justification of Insulation Thickness and Wall Assembly

**Response:** Thank you for raising this important point.

 Regarding thickness, the selected values were not arbitrary. The 75 mm mycelium thickness corresponds to the commercial BIOHM® panel used in the simulation. The 70 mm XPS and 50 mm Rockwool thicknesses were adopted from a comparable DesignBuilder study of residential buildings in Cairo, which used these exact thicknesses for insulation materials available in the Egyptian market (Elkhayat et al., 2023). The thermophysical properties of XPS and Rockwool were additionally based on data from Rockal Al Alamia, an Egyptian insulation manufacturer. Thus, the selected configurations were intended to represent realistic applications in the Egyptian context rather than an equal-thickness comparison.

 Regarding the wall assembly, the positioning also follows Elkhayat et al. (2023), where 50 mm Rockwool was applied toward the exterior of the masonry wall, while 70 mm XPS was incorporated within a double-leaf brick wall. The external positioning of Rockwool is also consistent with the External Thermal Insulation Composite System (ETICS) approach, in which mineral-wool insulation boards are installed on the external wall substrate and protected by a reinforced render/finishing system (EOTA, 2025; ROCKWOOL, n.d.).

 We acknowledge that differences in insulation thickness and layer position can influence thermal performance. Therefore, the results should be interpreted as a comparison of representative material configurations, rather than a controlled equal-thickness or equal-R-value comparison.

**References**

 Elkhayat, Y. O., Elmozy, S. A., & El-Darwish, I. I. (2023). Selecting the optimal thermal insulation material for a residential building envelope in Cairo in consideration of the thermal comfort and energy consumption. *Journal of Sustainable Architecture and Civil Engineering, 2*(33), 122–132. https://doi.org/10.5755/j01.sace.33.2.32751

**European Committee for Standardization (CEN). (2003).** *EN 13500:2003: Thermal insulation products for buildings, External thermal insulation composite systems (ETICS) based on mineral wool,Specification.* Brussels: European Committee for Standardization

# Response

**Published:** 2026-09-02

**Authors:** passant khaled

**Affiliations:** Interior, Arab Academy for Science Technology and Maritime Transport College of Engineering and Technology, Alexandria, Alexandria Governorate, Egypt

**Response – Small Performance Difference Between Mycelium and XPS**

**Response:** Thank you for this comment.

 The small difference in energy reduction between Mycelium (15.8%) and XPS (15.7%) was not intended to suggest that Mycelium outperforms XPS. Rather, the results indicate that the two materials provide **comparable thermal and energy performance**, as the 0.1 percentage-point difference is not considered practically significant.

 This comparability is an important finding of the study: Mycelium achieved similar simulated performance to the conventional XPS insulation. Therefore, our conclusion is not that Mycelium performs better than XPS, but that it represents a **potential alternative with comparable simulated thermal performance**. Its additional environmental characteristics, such as biodegradability and potential for lower embodied environmental impacts, are based on findings reported in the literature rather than quantified through the present simulation.

# Response

**Published:** 2026-09-02

**Authors:** passant khaled

**Affiliations:** Interior, Arab Academy for Science Technology and Maritime Transport College of Engineering and Technology, Alexandria, Alexandria Governorate, Egypt

**Environmental Claims / LCA, Cost and Durability**

**Response:** Thank you for this comment.

 The scope of this study is limited to the **thermal and energy performance** of the insulation alternatives; therefore, a full life-cycle assessment (LCA), cost analysis, and durability assessment were not conducted.

 The environmental characteristics of Mycelium discussed in the manuscript, including embodied carbon and biodegradability, are **literature-based values rather than findings generated by the present simulation**. These data are separately presented in Table 10 with their respective literature sources, while the findings of this study are based on the simulated U-value, PMV/PPD, discomfort hours, and energy consumption.

 Accordingly, the sustainability discussion is intended to provide context for the simulated thermal results rather than to represent a quantified environmental assessment conducted within this study. A comprehensive LCA, economic assessment, and long-term durability evaluation have already been identified under **Further Research** as necessary next steps to assess the broader feasibility of Mycelium insulation.

# Reviewer report: Reviewer response for version 1

**DOI:** 10.5256/f1000research.179279.r392628

**Published:** 2025-08-09

**Authors:** Jia Heng Teoh

**Affiliations:** 1 Nanyang Technological University (Ringgold ID: 54761), Singapore, Singapore

In the study titled, “Evaluating mycelium as an insulation material: A comparative study on thermal performance, comfort, and energy efficiency”, Khaled, Hany and Mossad have evaluated the use of mycelium-based materials as an insulation material in Egypt through simulations using a commercial software. Their studies have shown that compared to two other conventional insulation materials (XPS and Rockwool), mycelium-based materials performed better than or at the very least comparable to XPS and Rockwool in metrics relating to thermal performance and energy efficiency.

 The following are my comments regarding the study.

1. Please double check the definition of mycelium stated at the beginning of the literature review. The description fits the fruiting body more than mycelium, the vegetative or root-like structure of the fungus.
2. How easily accessible is mycelium in the area of the case study? What is the feasibility of fabricating mycelium-based material using locally available resources in Egypt? While mycelium-based materials are sustainable, if mycelium and the substrate need to be imported, the carbon footprint and energy consumed in transportation will negatively affect its sustainability.
3. Regarding the mycelium-based material that is being simulated in this study, what is the fungal species and the substrate or agricultural waste being assumed in this study? Literature has shown that mycelium-based material can be made with a variety of fungal species and substrates and as such, a bit more specificity is required. 
4. Please include references to justify that XPS and Rockwool are indeed traditional insulation materials commonly used in Egypt. This is unclear to readers who are not based in Egypt. 

Is the case presented with sufficient detail to be useful for teaching or other practitioners?

Yes

Is the work clearly and accurately presented and does it cite the current literature?

Yes

If applicable, is the statistical analysis and its interpretation appropriate?

Yes

Are all the source data underlying the results available to ensure full reproducibility?

Yes

Are the conclusions drawn adequately supported by the results?

Yes

Is the background of the case’s history and progression described in sufficient detail?

Partly

Reviewer Expertise:

Advanced fabrication and characterization of mycelium-bound composites and fungal-based engineered living materials.

I confirm that I have read this submission and believe that I have an appropriate level of expertise to confirm that it is of an acceptable scientific standard, however I have significant reservations, as outlined above.

# Response

**Published:** 2025-12-12

**Authors:** passant khaled

**Affiliations:** Interior, Arab Academy for Science Technology and Maritime Transport College of Engineering and Technology, Alexandria, Alexandria Governorate, Egypt

**Reviewer comment 1:**

*“Please double check the definition of mycelium stated at the beginning of the literature review. The description fits the fruiting body more than mycelium, the vegetative or root-like structure of the fungus.”*

**Author response:**

 Thank you for this helpful observation. The definition of mycelium in the literature review has been corrected to accurately describe mycelium as the vegetative, filamentous network (hyphae) of a fungus rather than the fruiting body.

**Reviewer comment 2:**

*“How easily accessible is mycelium in the area of the case study?*

**Author response:**

 We appreciate this important point. A new paragraph has been added to the manuscript describing the current accessibility of mycelium in Egypt and the feasibility of local production. The text highlights emerging companies such as Cupmena and Mycelium Egypt, which cultivate mycelium using agricultural waste, demonstrating that mycelium-based materials can be produced locally without relying on imports. This addresses the sustainability concern and clarifies the material’s local availability.

# Reviewer report: Reviewer response for version 1

**DOI:** 10.5256/f1000research.179279.r392627

**Published:** 2025-08-08

**Authors:** Olga Beatrice Carcassi

**Affiliations:** 1 Columbia University, New York, New York, USA

**General Comments:**

 The paper provides a timely and relevant comparison between conventional insulation materials (XPS, Rockwool) and a bio-based alternative—mycelium. The use of Egyptian housing as a case study adds valuable regional relevance and could offer meaningful insights into sustainable construction practices in hot climates.

 However, while the research addresses an important topic and offers promising insights, the current version suffers from issues related to clarity, limited referencing, insufficient justification of methodology choices, and a lack of strong data to support its conclusions. Below are detailed comments categorized for clarity.

**1. Clarity, Structure, and Referencing**

- **Clarity:** The paper would benefit from substantial editing for overall coherence. Key sections (especially the background and conclusion) are unclear or contain vague statements that need further development and evidence by expanding the literature review
- **Referencing:** The literature review and referencing are insufficient.
  - Ref [1] is overused throughout the paper. It is essential to engage with a broader range of literature.
  - **Recommendation:** In the Introduction and Background sections, aim for at least one reference per factual statement, ideally two. This should also apply to the Conclusion section.
  - Include mycelium-specific references. The field has a rapidly growing body of literature, including studies on thermal conductivity, lifecycle assessment, biodegradability, and production methods.
  - Avoid generalizations without backing data. Each claim, especially those that compare performance or environmental impact, must be supported with references.

**2. Figures and Visuals**

- **Figure 1:** Please add the source of the data presented in this figure. It is crucial to ensure transparency in data visualization.
- **Figure 3:** This figure needs graphical improvement.
  - Ensure the copyright of any images used is properly cited or that permission has been obtained.
  - If images were generated using AI, please check the journal's policy on the use of AI tools for figures.
- **Figure 4:** Improve its readability, especially for readers unfamiliar with climate-responsive design tools.
  - Make clear if the legend relates to lines or data points.
  - Use color-coded arrows or graphical elements to help explain the design strategies.
  - The link between the figure and the strategies described below it must be visually reinforced (e.g., using matching colors or icons).

**3. Methodology and Assumptions**

- **Material Thickness Choices:** The rationale behind selecting different thicknesses for mycelium (75mm), XPS (70mm), and Rockwool (50mm) is unclear.
  - Since all three materials are commercially available at 75mm, a uniform comparison would be more appropriate.
  - Alternatively, if you're comparing them based on other premises, this needs to be explicitly stated and justified.
- **Wall Assembly Logic:**
  - The placement of mycelium in the middle layer and Rockwool on the outer layer must be explained.
  - Is this arrangement based on established construction practices? If so, please cite relevant references.
  - Otherwise, clarify the reasoning and whether this setup could realistically be implemented in Egyptian construction.

**4. Claims That Require Supporting Data**

- **Carbon Absorption Statement:**
*“Mycelium has the capability to capture and store over 16 metric tons of carbon in just one month.”*
  - This is an extremely bold claim. You must specify:
    - How much mycelium biomass is required to achieve this carbon sequestration?
- **Conclusion Section:**
  Several claims lack supporting references, such as:
  - XPS creating sealed environments prone to mold.
  - Biodegradability and fire risks of XPS.
  - Indoor air quality degradation.
  - Health impacts on building occupants.
  - **Recommendation:** Every statement in this section must be supported by peer-reviewed or technical reference. Avoid repeating unverified assumptions.
- **Mycelium Advantages (Bullet List):**
  - The following claims need references:
    - Lower cost – please include economic comparison per square meter using current market prices.
    - Biodegradability – supported by LCA or composting data.
    - Low energy in production – cite data comparing embodied energy of production.
    - Limited production locations – quantify or describe this constraint and its implications.
  - Overall, the conclusion needs to be grounded in stronger and more comprehensive data.

**5. Broader Argument and Contribution**

 Currently, the paper shows that mycelium is roughly comparable to XPS in performance. However, it does not clearly build a compelling case for why the construction sector should adopt it. For instance:

- Is it more sustainable across the entire lifecycle?
- Is it more cost-effective?
- Is there a regulatory or design advantage?

Without these arguments being data-driven, the conclusion remains speculative. The paper should include a stronger comparative synthesis, perhaps a table summarizing key performance indicators (e.g., thermal conductivity, embodied energy, biodegradability, toxicity, fire safety, cost).

**Final Recommendation:**

 The paper addresses an important topic with promising potential. However, due to issues with clarity, insufficient referencing, methodological gaps, and weak evidence in support of key claims, I recommend a decision once the author makes the necessary changes:

- Expand and strengthen the literature review,
- Justify modeling choices more rigorously,
- Substantiate all environmental and performance claims with references,
- Improve figure clarity and citation, the paper could make a valuable contribution to the field of sustainable construction materials.

Is the case presented with sufficient detail to be useful for teaching or other practitioners?

Partly

Is the work clearly and accurately presented and does it cite the current literature?

No

If applicable, is the statistical analysis and its interpretation appropriate?

Partly

Are all the source data underlying the results available to ensure full reproducibility?

Yes

Are the conclusions drawn adequately supported by the results?

Partly

Is the background of the case’s history and progression described in sufficient detail?

No

Reviewer Expertise:

Mycelium materials. Life-cycle assessment and thermal performance

I confirm that I have read this submission and believe that I have an appropriate level of expertise to confirm that it is of an acceptable scientific standard, however I have significant reservations, as outlined above.

# Response

**Published:** 2025-12-12

**Authors:** passant khaled

**Affiliations:** Interior, Arab Academy for Science Technology and Maritime Transport College of Engineering and Technology, Alexandria, Alexandria Governorate, Egypt

**Reviewer comment:**

*The paper does not clearly build a compelling case for why the construction sector should adopt mycelium. Several claims in the conclusion lack supporting references*

**Author response:**

 Thank you for this comment. The conclusion has been strengthened by adding additional peer-reviewed and technical references to support key claims. In addition, a new comparative table has been introduced to directly compare mycelium and XPS across indicators of thermal performance, embodied energy, emissions, biodegradability, fire safety, and overall environmental impact, based on published literature.
